# Induction, Multiplication, and Evaluation of Antioxidant Activity of *Polyalthia bullata* Callus, a Woody Medicinal Plant

**DOI:** 10.3390/plants9121772

**Published:** 2020-12-14

**Authors:** Munirah Adibah Kamarul Zaman, Azzreena Mohamad Azzeme, Illy Kamaliah Ramle, Nurfazlinyana Normanshah, Siti Nurhafizah Ramli, Noor Azmi Shaharuddin, Syahida Ahmad, Siti Nor Akmar Abdullah

**Affiliations:** 1 Department of Biochemistry, Faculty of Biotechnology and Biomolecular Sciences, Universiti Putra Malaysia, Selangor, Seri Kembangan 43400, Malaysia; gs49877@student.upm.edu.my (M.A.K.Z.); 184942@student.upm.edu.my (I.K.R.); gs56229@student.upm.edu.my (N.N.); gs52490@student.upm.edu.my (S.N.R.); noorazmi@upm.edu.my (N.A.S.); syahida@upm.edu.my (S.A.); 2 Institute of Plantation Studies, Universiti Putra Malaysia, Selangor, Seri Kembangan 43400, Malaysia; snaa@upm.edu.my; 3 Department of Agriculture Technology, Faculty of Agriculture, Universiti Putra Malaysia, Selangor, Seri Kembangan 43400, Malaysia

**Keywords:** *Polyalthia bullata*, callus induction, callus multiplication, auxins, total phenolic content, total flavonoid content, antioxidant activity

## Abstract

*Polyalthia bullata* is an endangered medicinal plant species. Hence, establishment of *P. bullata* callus culture is hoped to assist in mass production of secondary metabolites. Leaf and midrib were explants for callus induction. Both of them were cultured on Murashige and Skoog (MS) and Woody Plant Medium (WPM) containing different types and concentrations of auxins (2,4-dichlorophenoxyacetic acid (2,4-D), α-naphthaleneacetic acid (NAA), picloram, and dicamba). The callus produced was further multiplied on MS and WPM supplemented with different concentrations of 2,4-D, NAA, picloram, dicamba, indole-3-acetic acid (IAA), and indole-3-butyric acid (IBA) media. The quantification of total phenolic content (TPC), total flavonoid content (TFC) and antioxidant capacity was further carried out on *P. bullata* callus, and the results were subjected to correlation analysis. Among the media, the WPM + 16.56 µM picloram (53.33 ± 22.06%) was the best for callus induction while MS + 30 µM dicamba was the best for callus multiplication. The TPC, TFC, and EC_50_ of DPPH scavenging activity were determined at 0.657 ± 0.07 mg GAE/g FW, 0.491 ± 0.03 mg QE/g, and 85.59 ± 6.09 µg/mL in *P. bullata* callus, respectively. The positive correlation between DPPH scavenging activity with TPC was determined at r = 0.869, and that of TFC was at r = 0.904. Hence, the *P. bullata* callus has an ability to accumulate antioxidants. It therefore can be a medium for secondary metabolites production.

## 1. Introduction

*Polyalthia bullata* is a medicinal plant that belongs to the genus *Polyalthia* and family Annonaceae. It is a shrub species that has a single stem with thin branches covered with golden hairs as described by Global Information Hub on Integrated Medicine (GlobinMed) [1]. The plant has been reported to contain bioactive compounds such as alkaloids and flavonoids, which display anticancer activities towards leukemia and breast cancer [2,3,4]. In some countries, particularly in Malaysia, Thailand, and Indonesia, the plant has been used as an herbal aphrodisiac by the local citizens, apart from treating asthma, diabetes, waist pain, skin problems, high blood pressure, diabetes, and liver disease [5,6,7]. These medicinal potentials have led to massive exploitation of this plant from wild habitats, which can later lead to species extinction.

The in vitro propagation technique has been proven to be one of the most powerful tools to mass produce and accelerate production of medicinal plants, food crops, and ornamental plants [8]. In addition, this approach can be a way for a large-scale production of seedlings or calluses to support commercial industries such as herbal, nutraceutical, and pharmaceutical industries [9].

Callus formation is one of the steps involved in in vitro plant propagation, and it has been used in both research and industrial applications. Several factors may influence the success of callus induction and multiplication of woody plants, which include the selection of suitable basal medium and plant growth regulators (PGRs). The correct concentration of PGRs is also crucial since different plant species require different conditions for callus growth [10,11,12]. Generally, the right ratio of exogenous auxin and cytokinin contributes to the formation of calluses. A high ratio of auxin to cytokinin or cytokinin to auxin into the callus medium typically facilitates root and shoot regeneration, while a balanced ratio of auxin and cytokinin promotes callus induction [13,14]. However, in some cases, application of auxin alone has also successfully assisted in callus induction and growth [13].

Auxins play a vital role in plant growth, cell expansion, cell division, and cell differentiation [15,16]. Auxins exist either as natural or synthetic analogs, which are frequently used in agriculture for plant development and improvement. Natural auxins like indole-3-acetic acid (IAA), phenyl acetic acid (PAA), and indole-3-butyric acid (IBA) are synthesized from plants or bacteria [17]. Naphthaleneacetic acid (NAA), 2,4-dichlorophenoxyacetic acid (2,4-D), 3,6-dichloro-2-methoxybenzoic acid (dicamba), and 4-amino-3,5,6-trichloropicolinic acid (picloram) are synthetic auxins that have an ability to act as natural auxins [18]. Some of these auxins play a crucial role in callus induction and proliferation, but the success rate is species dependent [19]. According to Osman et al. [11], 2,4-D has been recognized as an auxinic compound, and it has been reported to successfully induce callus formation in various plant species due to its ability to activate the cell division process in plants. Other auxins like picloram can activate the stress response that promotes cell dedifferentiation and induces callus formation when applied at high concentration [20].

Phenolics and flavonoids are compounds that have been used as food additives, flavors, and ingredients in cosmetics products [21]. These compounds are also involved in preventing various diseases due to their antioxidant, anti-cancer, and anti-inflammatory properties [22]. Antioxidants are defined as substances that have an ability to stabilize the reactive oxygen species (ROS) and other oxidants [23]. For instance, polyphenol compounds such as resveratrol and curcumin are known for their antioxidant properties, and they have been used to treat many ailments [24,25]. In comparison to resveratrol and curcumin, the polyphenol ellagic acid has been used in the food industry. Ellagic acid is a strong antioxidant, which can preserve food quality due to its ability to scavenge numerous ROS and prevent lipid peroxidation in food products [26]. In addition, epigallocatechin gallate, the abundant catechin in green tea has been reported to possess scavenging activity towards ROS due to its ability to act as an electron donor and ability to chelate metal ions [27]. The callus culture can be a source of antioxidant production, and the technique has been used as an alternative for the production of plant secondary metabolites. The callus with a greater biomass is the best for extraction of secondary metabolites, and therefore searching out the best medium for each plant species is important [28].

In this study, we aimed to determine the effect of culture medium, and types and concentrations of auxins on callus induction and multiplication of the woody plant, *P. bullata*. The resulting callus was further subjected for TPC, TFC, and 2,2′-diphenyl-1-picrylhydrazyl (DPPH) scavenging activity analyses.

## 2. Results

### 2.1. Effect of Different Types and Concentrations of Auxins on Callus Induction and Morphology

Table 1 displays responses of different explants when they were cultured on Murashige and Skoog (MS) containing 2,4-D, NAA, and picloram, respectively at different concentrations under light and dark conditions. Among the treatments, midribs incubated in the dark on MS + 16.56 µM picloram induced the highest callus (31.11 ± 12.86%) after 66 days of culture. The leaf explants cultured at adaxial and abaxial positions on all MS combination media, however, failed to induce callus formation; therefore, the results are not presented in Table 1. A negative response was also observed on callus that was cultured on MS basal media without auxins (MSO) and MS supplemented with different concentrations of dicamba under light and dark conditions.

The use of woody plant medium (WPM) with different concentrations and types of auxins showed that dicamba was not the best auxin to induce calluses from all *P. bullata* explants (Table 2). The negative response was also observed for WPMO medium where no callus was produced from all explants and both conditions. However, when compared with MS, the midrib cultured on WPM + 16.56 µM picloram in dark produced the highest callus at 53.33 ± 22.06% after 78 days of culture.

Different morphological types of callus were obtained from leaf and midrib explants cultured on MS and WPM media supplemented with different types and concentrations of auxins. In terms of callus morphology, the callus induced from explants cultured on media supplemented with picloram and NAA produced friable callus while explants cultured on media supplemented with 2,4-D showed compact callus.

### 2.2. Effect of Auxin Supplemented MS and WPM Media on Callus Growth and Multiplication

**Figure 1:**
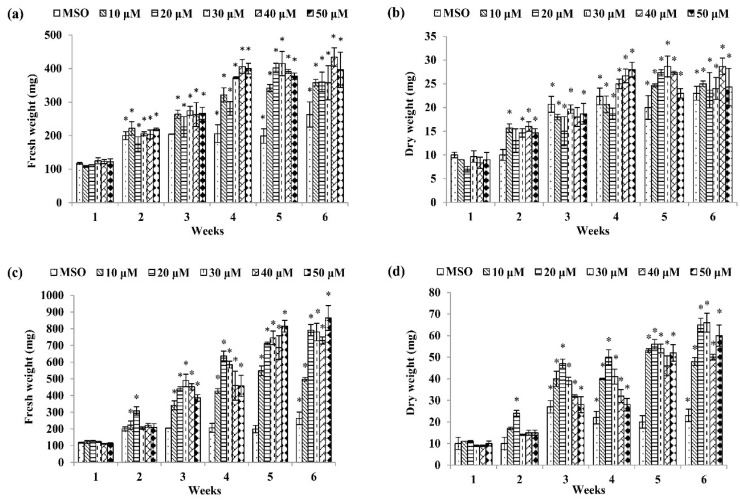

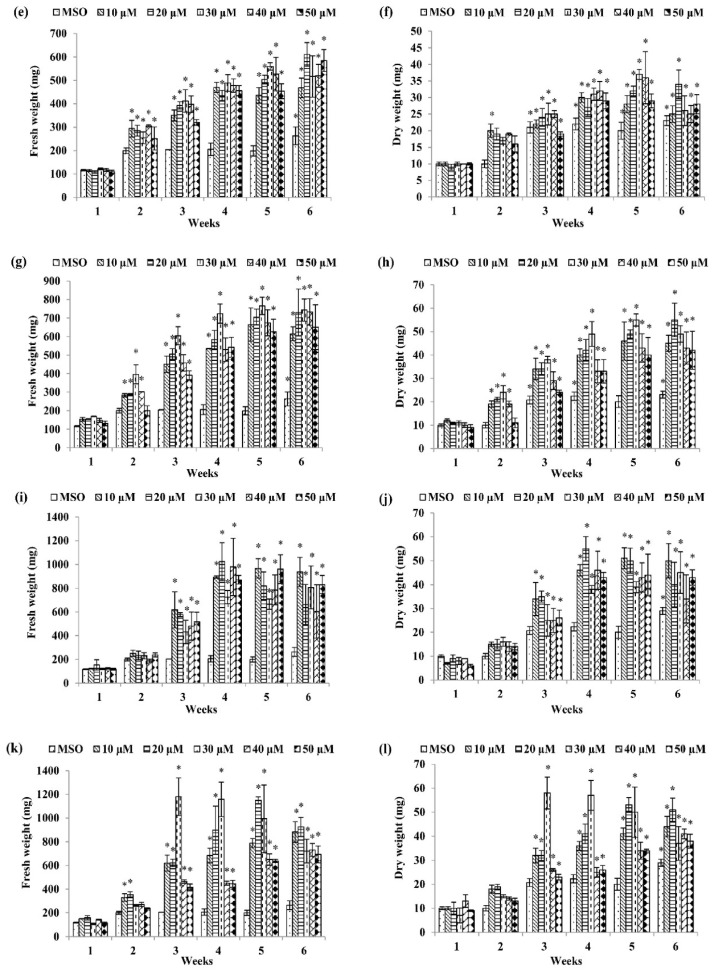
Fresh weight and dry weight of *P. bullata* callus grown on MS medium at different concentrations of (**a**,**b**) α-naphthaleneacetic acid (NAA), (**c**,**d**) indole-3-butyric acid (IBA), (**e**,**f**) indole-3-acetic acid (IAA), (**g**,**h**) 2,4-D, (**i**,**j**) picloram, and (**k**,**l**) dicamba. Data represented as means ± SE. The asterisk (*) represents a significant difference of treated callus with 1-week old callus grown on MS basal media without auxins (MSO) at *p* ≤ 0.05 Tukey’s range test.

**Figure 2:**
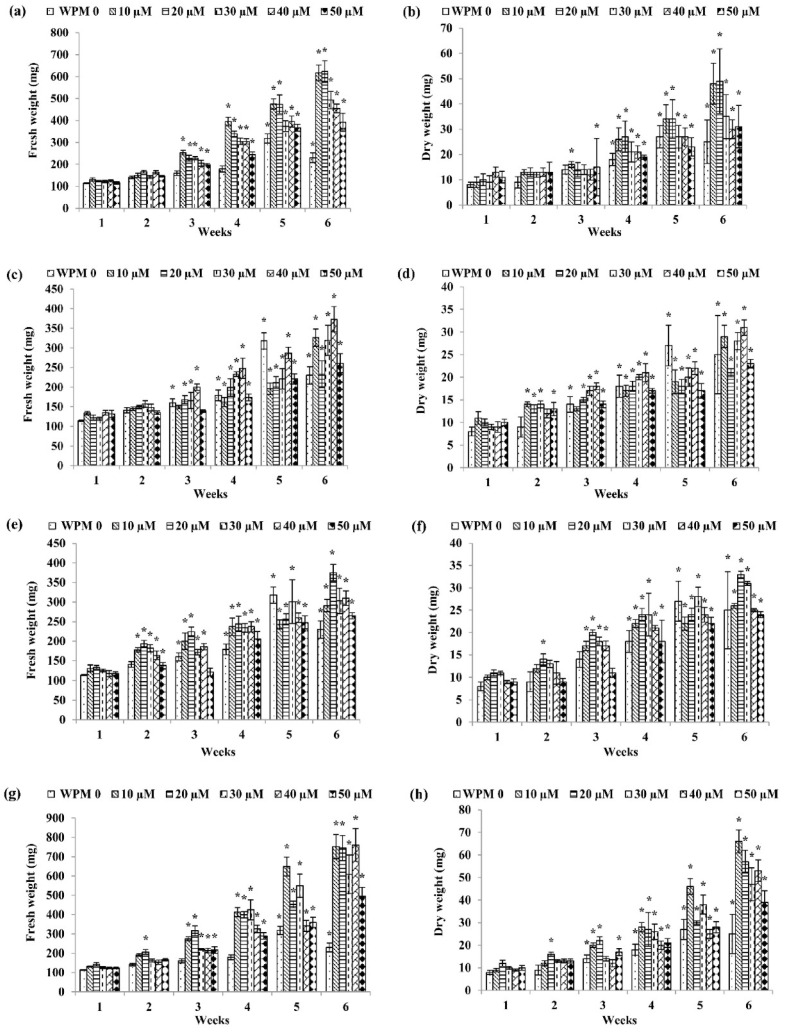

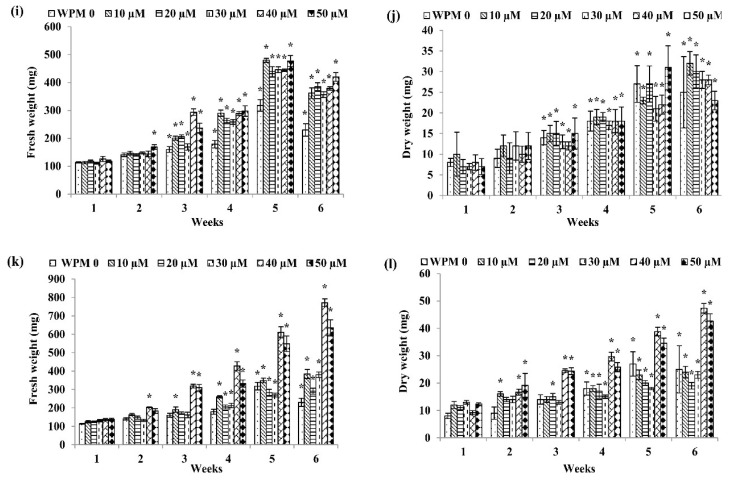
Fresh weight and dry weight of *P. bullata* callus grown on WPM medium at different concentrations of (**a**,**b**) NAA, (**c**,**d**) IBA, (**e**,**f**) IAA, (**g**,**h**) 2,4-D, (**i**,**j**) picloram and (**k**,**l**) dicamba. Data represented as means ± SE. The asterisk (*) represents a significant difference of treated callus with 1-week old callus grown on WPM basal media without auxins (WPMO) at *p* ≤ 0.05 Tukey’s range test.

Among the basal media tested, a combination of MS and auxins was the best to induce callus formation of *P. bullata* (Figure 1) compared to a combination of WPM and auxins (Figure 2). The MS medium supplemented with 30 µM dicamba exhibited the highest fresh weight (1180.00 ± 159.43 mg FW) (Figure 1k) after three weeks of culture followed by MS + 20 µM picloram after four weeks of culture (1024.00 ± 158.15 mg FW) (Figure 1i), MS + 50 µM IBA after six weeks of culture (864.00 ± 74.84 mg FW) (Figure 1c), MS + 30 µM 2,4-D after five weeks of culture (766.00 ± 46.66 mg FW) (Figure 1g), MS + 20 µM IAA after six weeks of culture (611.00 ± 50.54 mg FW) (Figure 1e), and MS + 40 µM NAA after six weeks of culture (434.33 ± 27.79 mg FW) (Figure 1a).

For dry weight (DW), the highest dry weight was shown by callus treated with 30 µM IBA (66.00 ± 4.37 mg DW) (Figure 1d) after six weeks of culture followed by MS + 30 µM dicamba after three weeks of culture (58.00 ± 6.66 mg DW) (Figure 1l), MS + 20 µM picloram after four weeks of culture (54.67 ± 5.04 mg DW) (Figure 1j), MS + 30 µM 2,4-D after five weeks of culture (55.00 ± 2.60 mg DW) (Figure 1h), MS + 30 µM IAA after five weeks of culture (37.00 ± 1.53 mg DW) (Figure 1f), and MS + 40 µM NAA after six weeks of culture (28.67 ± 1.76 mg DW) (Figure 1b).

As for WPM media (Figure 2), the highest fresh weight (FW) was recorded in the callus treated with 40 µM dicamba with 771.50 ± 20.86 mg FW after six weeks of culture (Figure 2k) followed by WPM + 40 µM 2,4-D after six weeks of culture (760.67 ± 85.21 mg FW) (Figure 2g), WPM + 20 µM NAA after six weeks of culture (623.67 ± 49.34 mg FW) (Figure 2a), WPM + 10 µM picloram after five weeks of culture (480.33 ± 18.94 mg FW) (Figure 2i), WPM + 20 µM IAA after six weeks of culture (375.33 ± 20.95 mg FW) (Figure 2e), and WPM + 40 µM IBA after six weeks of culture (373.33 ± 32.36 mg FW) (Figure 2c).

For dry weight, callus grown on WPM + 20 µM 2,4-D showed the highest dry weight after six weeks of culture (66.17 ± 5.09 mg DW) (Figure 2h) followed by callus grown on WPM + 20 µM NAA after six weeks of culture (49.17 ± 64.26 mg DW) (Figure 2b), WPM + 40 µM dicamba after six weeks of culture (47.33 ± 1.73 mg DW) (Figure 2l), WPM + 20 µM IAA after six weeks of culture (32.83 ± 1.47 mg DW) (Figure 2f), WPM + 10 µM picloram after six weeks of culture (32.17 ± 61.81 mg DW) (Figure 2j), and WPM + 40 µM IBA after six weeks of culture (31.33 ± 1.69 mg DW) (Figure 2d).

**Figure 3:**
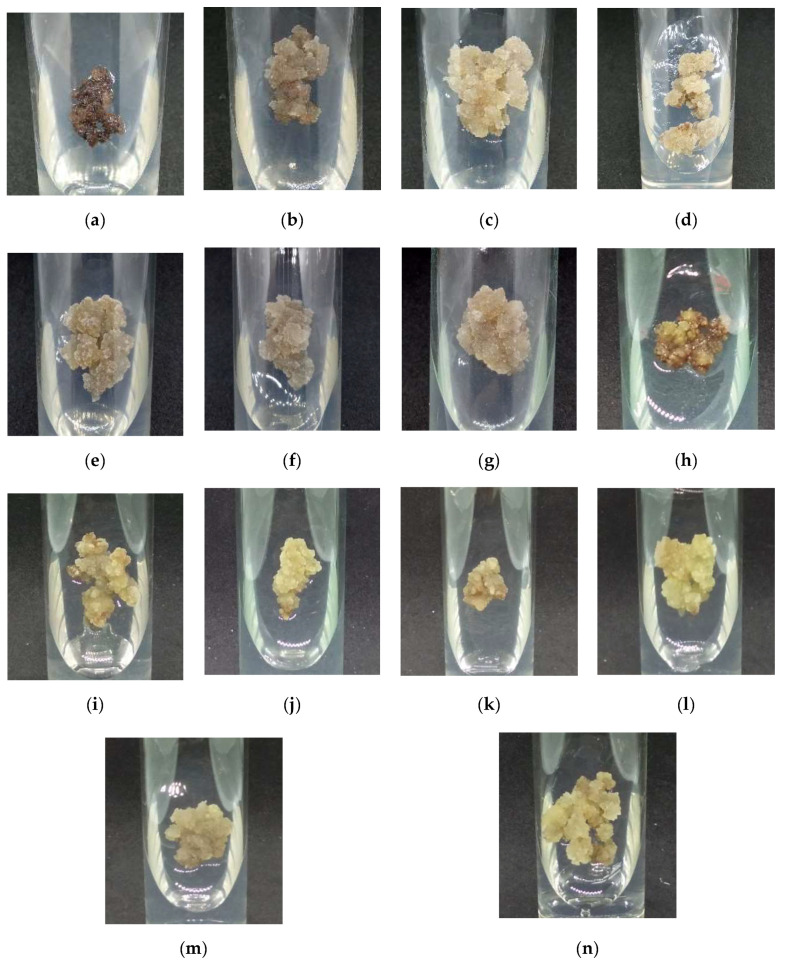
Morphology of callus cultured on MS and WPM media supplemented with (**a**) MSO, (**b**) MS + 40 µM NAA, (**c**) MS + 50 µM IBA, (**d**) MS + 20 µM IAA, (**e**) MS + 30 µM 2,4-D, (**f**) MS+ 20 µM picloram, (**g**) MS + 30 µM dicamba, (**h**) WPMO, (**i**) WPM + 20 µM NAA, (**j**) WPM + 40 µM IBA, (**k**) WPM + 20 µM IAA, (**l**) WPM + 40 µM 2,4-D, (**m**) WPM + 10 µM picloram, and (**n**) WPM + 40 µM dicamba after six weeks of cultivation in dark condition at 25 ± 2 °C.

The morphology of callus cultured on MS and WPM medium supplemented with auxins is shown in Figure 3. Compact callus was produced from callus grown on MSO (Figure 3a) and WPMO (Figure 3h). Friable callus was produced from MS + 50 µM IBA- (Figure 3c), MS + 20 µM picloram- (Figure 3f), and MS + 30 µM dicamba-treated callus (Figure 3g). Meanwhile, MS + 40 µM NAA- (Figure 3b), MS + 20 µM IAA- (Figure 3d), and MS + 30 µM 2,4-D-treated callus (Figure 3e) produced mixed callus (friable and compact callus). The production of mixed callus was also observed from callus grown on WPM + auxins (Figure 3 i–n).

Overall, the callus treated with MS + 30 µM dicamba showed the highest callus biomass (Figure 1k) with the friable callus (Figure 3g) as compared with the other treatments.

### 2.3. Determination of Total Phenolic (TPC) and Total Flavonoid (TFC) Content

The callus grown on MS + 30 µM dicamba at week 3 was further selected for determination of TPC and TFC since the treatment showed the highest callus biomass with friable callus as compared to the other treatments. The TPC and TFC of callus treated with MS + 30 µM dicamba are shown in Table 3.

### 2.4. Determination of Antioxidant Activity

**Figure 4:**
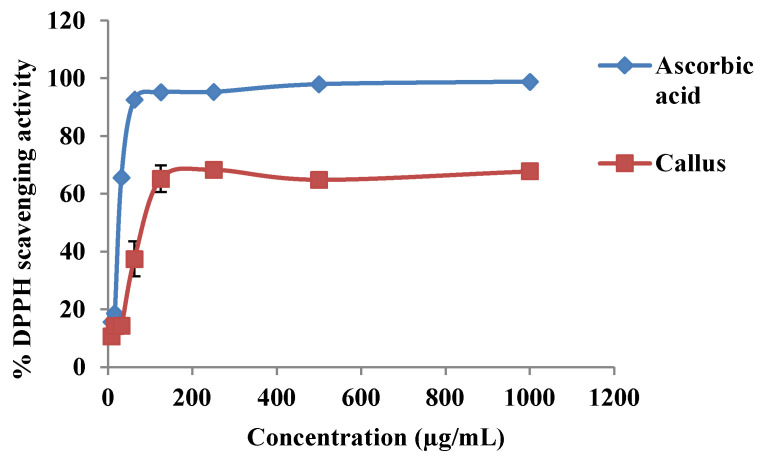
The 2,2′-diphenyl-1-picrylhydrazyl (DPPH) scavenging activity at different concentrations of ascorbic acid and methanolic extract of *P. bullata* callus.

**Figure 5:**
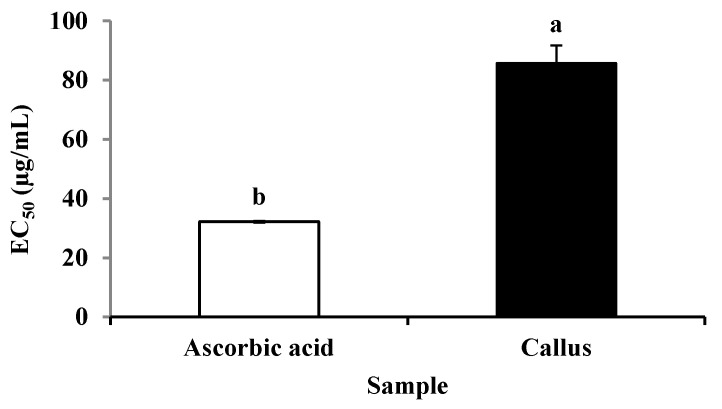
The EC_50_ value of DPPH scavenging activity of ascorbic acid and methanolic extract of *P. bullata* callus. The results expressed as the means ± standard error (n = 3). Means with different letters indicate significant difference at *p* ≤ 0.05.

There was an increase in DPPH scavenging activity with an increase in callus extract and ascorbic acid concentrations (Figure 4). The DPPH scavenging activities between ascorbic acid and callus extract were also expressed as 50% effective concentration (EC_50_) as shown in Figure 5. The EC_50_ values of callus extract and ascorbic acid were recorded at 85.59 ± 6.09 µg/mL and 32.16 ± 0.29 µg/mL, respectively. The result was also expressed as ascorbic acid equivalent antioxidant capacity, and the value obtained was 1.879 ± 0.22 mg AEAC/g FW of extract.

### 2.5. Correlation between TPC, TFC, and Antioxidant Activity of P. bullata Callus

The relationships between antioxidant activity with TPC and TFC are shown in Table 4. The positive correlations obtained between DPPH scavenging activity with TPC and TFC were at *r* = 0.869 and *r* = 0.904, respectively. The TFC showed the highest correlation with DPPH scavenging activity as compared to TPC in methanolic extract of *P. bullata* callus.

## 3. Discussion

Auxins are the best PGRs to enhance chances of callus formation [29,30]. Among the different auxins used, picloram and 2,4-D were the auxins that were successfully induced callus from leaf and midrib explants of *P. bullata* followed by NAA. The treatment with dicamba, however, failed to induce callus. The callus formed in picloram containing medium was friable. Meanwhile, the compact callus was produced on 2,4-D containing medium, and the friable callus was produced from NAA containing medium. The difference in callus formation and its morphology are due to the variation in physiological activity of the respective auxin within the plant tissue [31]. For instance, the friable callus was obtained from MS medium containing 2,4-D, and compact callus was obtained from MS containing NAA and IBA of *Ficus religiosa* culture [32].

In the present study, auxin herbicide picloram was the best auxin to induce callus due to toxicity effects of other auxins towards the plant cell. Kumar et al. [33] and Moura et al. [34] reported similar studies, in which picloram showed a better callus induction in *Triticum aestivum* L. and *Eucalyptus grandis* x *E. urophylla*. In contrast, a study by Binte Mostafiz and Wagiran [19] showed that picloram displayed the lowest callus induction percentage compared to dicamba and 2,4-D in Indica rice MR219. A study by Akram and Aftab [35] found that the addition of picloram into the culture medium failed to induce callus from *Tectona grandis* L.

All explants cultured on MSO and WPMO failed to induce callus both in light and dark conditions. This finding further strengthens the positive effects of auxins towards callus induction of *P. bullata*. The present study showed wounding followed by incubation of explants on high concentrations of auxin helped in the formation of *P. bullata* callus suggesting the involvement of regulatory cascades and pathways from the auxin-based route and the wound-induced route. The results obtained from this study were in accordance with the earlier reports by Osman et al. [11] and de Souza et al. [36], where no callus was induced from *Barringtonia racemosa* and *Genipa* sp. explants cultured on MSO and WPMO media. In addition, the MSO medium failed to induce callus from *Sargassum polycystum* explants [37]. 

Explants from *P. bullata* midrib were better at inducing callus compared to the leaf explants cultured at abaxial and adaxial positions. It suggests different cellular dedifferentiation capability between the two explants in producing callus [14]. The present study was in line with previous findings reported by Naz and Khatoon [38], Singh et al. [39], and Lamaoui et al. [40] on the effectiveness of *Achyranthes aspera*, *Sapindus mukorossi*, and *Argania spinosa* midribs in callus induction, respectively.

Two growth conditions, the dark and light, were also tested for callus induction of *P. bullata*, and the results showed the dark condition was more favorable for this plant. A similar result was obtained by Habibah et al. [41]. Their findings showed that the incubation of *Stelechocarpus burahol* explants exhibited the highest callus formation in the dark compared to the light condition. *S. burahol* is a tropical species, which can be found in Java, Indonesia. The plant likes hot and humid secondary forest on deep soil that is dominated with moist clay soils. It can grow up to 25 m tall with a trunk diameter of up to 40 cm [42]. A study by Adil et al. [43] also obtained maximum callus fresh weight of *Cnidium officinale* grown in completely dark condition. The *C. officinale* is a perennial plant from the family Umbelliferae or Apiaceae that is mainly cultivated in Korea, China, and Japan for its condiment and medicinal uses [43,44]. Hence, the dark condition was found to improve callus formation, which might be due to the suppression of photooxidative damage [45].

Between the different media tested, WPM medium was found to be the best basal medium to induce callus of *P. bullata*. The differences in the content of macronutrients and micronutrients presented in the MS and WPM media might influence the induction of *P. bullata* callus. A study by Osman et al. [11] found that the nutrient compositions in the culture medium might affect the responses of explants towards the formation of callus. There are certain nutrients present in WPM medium but not in the MS medium such as potassium sulfate, ferrous sulphate heptahydrate, and calcium nitrate that might be directly involved in callus induction of woody plants like *P. bullata*. Moreover, there is also a great difference in the amount of nutrients available in MS medium such as ammonium nitrate and calcium chloride (1650 mg/L and 440 mg/L, respectively) compared to that of WPM medium (400 mg/L and 72.5 mg/L, respectively). The high concentration of ammonium salts in the MS medium might influence the formation of callus in *P. bullata*. This has been proved by Behbahani et al. [45] where they found that the high concentration of ammonium salt could slow the callus formation since the ammonium salt can act as a growth inhibitor for plant tissue.

The callus growth curve provides quantitative data to identify the stages or phases of callus growth. The curve is important to determine the best time to subculture the callus into a new medium [46]. There are several stages of plant development which are lag, exponential, linear, stationary, and decreasing phases [47]. The lag phase indicates the starting of metabolite and protein mobilization. Meanwhile, the exponential phase shows the maximum cellular division. The callus needs to be transferred into a new medium due to the depletion of nutrients, agar dryness, as well as accumulation of toxic substances. Meanwhile, at the stationary phase, no weight increase of culture occurs, but the maximum accumulation of secondary metabolites occurs. Cellular death happens during the decline phase [46,48].

The presence of auxins enhanced the proliferation and growth of *P. bullata* callus. Dicamba produced the highest callus weight at the third week, which was earlier compared to 2,4-D and picloram. This is due to the already reported ability of dicamba to initiate the growth of callus even after two to three days of culturing on nutrient medium [49]. The use of dicamba as a single PGR in research on *Centella asiatica* was reported to be effective in promoting callus growth [9]. The *C. asiatica* is a tropical medicinal plant from the Apiaceae family, native to Southeast Asian countries such as India, Sri Lanka, China, Indonesia, and Malaysia as well as South Africa and Madagascar [50]. This plant grows in swampy areas of tropical and subtropical regions of the world [51]. According to Malik et al. [49], the rate of callus growth induced by dicamba increased with the increase of its concentration. This is contrary to the present study since the higher concentrations of dicamba at 40 and 50 μM reduced the growth of *P. bullata* callus. On the other hand, Behrens et al. [52] reported that most dicotyledonous plants, like tobacco, are quite sensitive to the application of auxin herbicide like dicamba. According to Bienaimé et al. [53], the presence of auxins is vital in maintaining cellular activity in callus as well as photosynthesis in the culture. The use of hormone free medium leads to necrosis of the callus cell.

The presence of phenolics and flavonoids was also quantified in the callus of *P. bullata*, which further proves the capability of leaf-derived (from leaf midrib) callus to produce antioxidant compounds. *P. bullata* leaf was recently reported to contain TPC and TFC as well as having high antioxidant capacity [54]. The TPC and TFC of the methanolic extract of *P. bullata* leaf were recorded at 1042.52 ± 1.97 mg GAE/g DW and 80.88 ± 0.24 mg QE/g DW, respectively, which was slightly higher than that of TPC and TFC in the *P. bullata* callus. The DPPH scavenging activity of *P. bullata* callus (67.79 ± 0.24%) was found to be lower than *P. bullata* leaf (85.19 ± 0.67%). Phenolics and flavonoids possess antioxidant properties by working as chain breakers, free radical scavengers and electron donors. The high amount of phenolics and flavonoids in the plant extract suggests a great radical scavenging and antioxidant capacity of the extracts [55]. A stable free radical, DPPH, was used to accept hydrogen atoms donated by antioxidants present in the extracts. This method is reliable due to its simplicity and ability in scavenging ROS [56,57]. The lower DPPH scavenging activity in callus as compared to ascorbic acid might be due to the low amount of phenolic and flavonoid compounds present in the callus extract compared to that of the pure compound. Similarly, a study by Esmaeili et al. [58] also found a lower DPPH scavenging activity in callus extract of *Trifolium pratense* as compared to ascorbic acid.

The EC_50_ value was defined as the antioxidant concentration required in obtaining a 50% radical inhibition [59]. In this study, the EC_50_ value obtained for callus extract was at 85.59 ± 6.09 µg/mL, which is significantly higher as compared to ascorbic acid (32.16 ± 0.29 µg/mL). According to Olugbami et al. [60], the lower the EC_50_ value, the higher the antioxidant activity of the extract. The higher EC_50_ was also found in methanolic extract of *T. pratense* callus (128.42 ± 2.40 µg/mL) as compared to a standard ascorbic acid (21.17 ± 0.76 µg/mL) [58]. A study by Umesh and Abhirami [61] also found a higher EC_50_ value in callus extract of *Asystasia gangetica* (65.775 ± 2.302 µg/mL) as compared to ascorbic acid (4.2096 ±.0891µg/mL).

The antioxidant activity of *P. bullata* callus was also expressed as ascorbic acid equivalent. This method was used to express the antioxidant activity for direct comparison with the well-known potent antioxidant that is widely distributed in plant materials [62]. The higher the ascorbic acid equivalent value, the higher the antioxidant activity [60]. The study by Tadhani et al. [63] found that the callus of *Stevia rebaudiana* exhibited a higher ascorbic acid equivalent with 32.32 ± 0.56 mg AEAC/g. The ascorbic acid equivalents from *Salvia tebesana* callus that were derived from different explants showed a variation of antioxidant activity ranging from 14.1 ± 0.32 to 36.2 ± 0.35 mg AEAC/100 g extract [64].

From the results obtained, the total phenolics and flavonoids showed a high correlation with the DPPH scavenging activity. In line with a previous study conducted by Krishnan et al. [65], the high correlations between DPPH with TPC and TFC were observed in *Gynura procumbens* callus at 0.762 and 0.691, respectively. Similar findings were also demonstrated by Kapoor et al. [66] where the TPC was significantly correlated with DPPH scavenging activity in callus of *Rhodiola imbricate*. It could be deduced that TPC and TFC contributed to the antioxidant activity in *P. bullata* callus.

## 4. Materials and Methods 

### 4.1. Plant Materials

The *P. bullata* plant is distributed as an understory tree or shrub, or as a main canopy tree in primary or secondary forests of lowland forests in peninsular Malaysia and Sabah. Sometimes it can be found in lower montane forests up to an altitude of 1200 m. It also grows well in evergreen and monsoon forests both on well-drained or poorly-drained terrains [67]. In this study the *P. bullata* plants were obtained from Herbal Nursery located at Pahang, Malaysia. The plants were then acclimatized in a greenhouse located at Universiti Putra Malaysia under ambient temperature for a month prior to analysis. The verification of *P. bullata* plants was done by Forest Research Institute Malaysia (FRIM). The plant identification number was given as PID 170820-13. The young *P. bullata* leaf with midrib, aged 2 months, was surface sterilized and aseptically cut into small pieces approximately 0.5 × 0.5 cm (leaf) and 0.3 cm (midrib). Both were cultured on MS [68] and WPM [69] containing different types and concentrations of auxins for callus induction.

### 4.2. Callus Induction

The sterile leaf and midrib (from Section 4.1) were cultured on MS [68] and WPM [69] supplemented with various concentrations of auxins: 2,4-D (9.05, 18.10 27.14, 36.19, 45.24 µM); NAA (10.74, 21.48, 32.22, 42.96, 53.70 µM); picloram (8.28, 16.56, 24.85, 33.13, 41.41 µM), and dicamba (9.05, 18.10, 27.14, 36.19, 45.24 µM) medium, respectively. The MS and WPM basal media without auxins named as MSO and WPMO were used as control throughout the studies. Nine explants were cultured on each medium with five replicates. One replicate contained nine leaf discs or midribs. The explants were then incubated at 25 ± 2 °C under light (16 h) and dark (8 h) conditions. The light source was produced by Tubular 8 (T8) day LED 1080 lumen. The callus induction percentage (%) was calculated according to the following formula:

$$Callus\;induction\;percentage\;\left(\%\right)=\;\frac{No.\;of\;explants\;cultured}{No.\;of\;explants\;produced\;callus}\;\times 100$$

### 4.3. Callus Multiplication and Determination of Callus Biomass and Morphological Changes

To determine the effect of auxins on callus multiplication, 100 mg of the calli obtained were cultured on MS and WPM media containing 10, 20, 30, 40, and 50 µM of 2,4-D, picloram, dicamba, IAA, IBA, and NAA, respectively. The calli grown on MS and WPM without addition of auxins served as controls. The cultures were then incubated at 25 ± 2 °C under dark conditions. The calli were grown in glass vials (25 mm × 100 mm), respectively. Each of the treatments consisted of six replicates.

The growth of calli was determined by measuring the calli fresh weight (FW) and dry weight (DW) every week for 6 weeks. The same calli used for fresh weight determination were later dried in the oven at 50 °C until they reached a constant weight for determination of callus dry weight. The morphological changes of callus in different treatments were also observed every week for 6 weeks.

### 4.4. Extraction of TPC and TFC from Callus, and Determination of Antioxidant Activity

The callus grown on MS + 30 µM dicamba at week 3 was chosen for determination of TPC, TFC, and antioxidant activity since it showed the highest callus biomass. The extraction of callus was conducted based on the method described by Giri et al. [70] with minor modification. The 200 mg fresh callus was weighed, ground using mortar and pestle, and extracted with 10 mL of 100% (*v/v*) methanol. The mixture was kept at room temperature with continuous shaking (150 rpm) for 24 hours. Then, the mixture was centrifuged at 8000 rpm for 10 minutes. The supernatant was collected and stored at 4 °C until further use. The samples were prepared in triplicate.

### 4.5. Determination of TPC

The TPC of the callus was determined by a method described by Osman et al. [71] with minor modification. The methanolic extract of *P. bullata* callus (1 mg/mL) as described in Section 4.4 was used for the analysis. The 0.5 mL of the sample was mixed with 2.5 mL of 10% Folin–Ciocalteu’s reagent and 2.5 mL of 7.5% sodium carbonate (NaCO_3_). The sample was incubated at room temperature for 90 min. The absorbance of the sample was determined using a spectrophotometer at 765 nm. The sample was prepared in triplicate, and the mean value of absorbance was obtained. The same procedure was repeated for the standard solution of gallic acid. The TPC was expressed as mg gallic acid equivalent per gram fresh weight (mg GAE/g FW).

### 4.6. Determination of TFC

The TFC of the callus was determined by a method described by Adhikari et al. [72] with minor modification. About 1 mL of the methanolic callus extract (as described in Section 4.4) was diluted to 5 mL with distilled water and mixed with 0.3 mL of 5% (w/v) sodium nitrite (NaNO_2_) solution. After 5 min, 0.3 mL of 10% (w/v) aluminum chloride (AlCl_3_) solution was added. Then, 2 mL of 1 M sodium hydroxide (NaOH) solution was added prior to addition of distilled water up to a final volume of 10 mL. The absorbance of the mixture was determined at 510 nm. A standard curve of quercetin was plotted. The analysis was conducted in triplicate. The TFC was expressed as mg of quercetin equivalents per gram of fresh extract (mg QE/g FW).

### 4.7. Antioxidant Assay

The antioxidant activity was determined using a 2,2-diphenyl-1-picryl-hydrazyl (DPPH) free radical scavenging activity assay adapted from Sumazian et al. [73] with minor modification. The initial absorbance of DPPH solution was measured without sample at 517 nm. Approximately 0.2 mL of each sample extract was mixed with 3 mL of 0.1 mM DPPH solution. The mixture was incubated at room temperature in the dark for 30 min. The change in absorbance was measured after 30 min of incubation at 517 nm using a UV-Visible spectrophotometer. Ascorbic acid was used as a standard. The result obtained was calculated and expressed in percent of DPPH free radical scavenging activity using the following formula,

$$\%\;\mathrm{of}\;\mathrm{DPPH}\;\mathrm{free}\;\mathrm{radical}\;\mathrm{scavenging}\;\mathrm{activity}\;=\;\frac{A\;control-A\;sample}{A\;control}\;\times \;100$$

where *A*_control_ is the absorbance of DPPH solution without sample, and *A*_sample_ is the absorbance of sample with DPPH solution.

The DPPH scavenging activity was analyzed at different concentrations of ascorbic acid and callus extract (7.81–1000 µg/mL). The DPPH scavenging activity was also expressed as the EC_50_ value (µg/mL) based on the linear regression of plots of mean percentage of the antioxidant activity against the concentration of the test extracts (μg/mL). The antioxidant activity was also expressed as ascorbic acid equivalent antioxidant capacity (AEAC) using the following formula: [60].

$$\frac{EC50\;ascorbic\;acid\;\left(\mathrm{mg}/\mathrm{mL}\right)}{EC50\;sample\;\left(\mathrm{mg}/\mathrm{mL}\right)}=\frac{\mathrm{mg}\;ascorbic\;acid\;equivalents}{\mathrm{mg}\;fresh\;weight}$$

where EC_50_ ascorbic acid and EC_50_ sample are the effective concentrations of ascorbic acid and sample, respectively.

### 4.8. Statistical Analysis

All the experiments were conducted based on the Completely Randomized Design (CRD). The data were analyzed using Proc analysis of variance (ANOVA) to compare the significant differences between treatments using Statistical Analysis Software (SAS 9.4). The comparison of means was determined by Tukey’s multiple range tests at *p* ≤ 0.05. The Pearson correlation coefficient was also performed using SAS 9.4 software. The EC_50_ value of the extract was calculated by GraphPad Prism 8 software.

## 5. Conclusions

In this report, the effectiveness of callus induction and multiplication of *P. bullata* depends greatly on the types of culture media as well as types and concentrations of auxins used. For callus induction, the WPM medium supplemented with 16.56 µM picloram in dark condition showed the highest callus induction percentage. Meanwhile, the callus cultured on the MS basal medium supplemented with 30 µM dicamba exhibited the best callus proliferation with the highest fresh weight and dry weight recorded at week three. The TPC and TFC from the callus were found to have a correlation with the DPPH scavenging activity. Hence, the establishment of *P. bullata* callus culture can be used as an approach to mass produce secondary metabolites, and therefore, may reduce overcollection of this plant in order to preserve its natural habitat as well as in other world areas.

## Figures and Tables

**Table 1:** Callus induction percentage of *P. bullata* midrib explants cultured on Murashige and Skoog (MS) basal medium treated with different types of auxins at different concentrations under light and dark conditions.

**Plant Growth Regulators (PGRs)**	**Light Condition**			**Dark Condition**		
	**Callus Induction Percentage (%)**	**Induction Time (Days)**	**Callus Morphology**	**Callus Induction Percentage (%)**	**Induction Time (Days)**	**Callus Morphology**
0	-	-	-	-	-	-
9.05 µM 2,4-D	-	-	-	-	-	-
18.10 µM 2,4-D	-	-	-	2.22 **^d^**	130	Compact
27.14 µM 2,4-D	2.22 **^d^**	111	Compact	-	-	-
36.19 µM 2,4-D	-	-	-	-	-	-
45.24 µM 2,4-D	-	-	-	2.22 **^d^**	94	Compact
8.28 µM picloram	11.11 **^cd^**	82	Friable	-	-	-
16.56 µM picloram	-	-	-	31.11 **^a^**	66	Friable
24.85 µM picloram	-	-	-	13.33 **^cd^**	66	Friable
33.13 µM picloram	2.22 **^d^**	67	Friable	28.89 **^bc^**	66	Friable
41.41 µM picloram	-	-	-	-	-	-
10.74 µM NAA	-	-	-	17.78 **^bcd^**	103	Friable
21.48 µM NAA	-	-	-	11.11 **^cd^**	101	Friable
32.22 µM NAA	-	-	-	15.56 **^bcd^**	98	Friable
42.96 µM NAA	-	-	-	4.34 **^d^**	96	Friable
53.70 µM NAA	2.22 **^d^**	90	Friable	-	-	-
9.05 µM dicamba	-	-	-	-	-	-
18.10 µM dicamba	-	-	-	-	-	-
27.14 µM dicamba	-	-	-	-	-	-
36.19 µM dicamba	-	-	-	-	-	-
45.24 µM dicamba	-	-	-	-	-	-

Different letters indicate the values of significant difference at *p* ≤ 0.05 Tukey’s range test for light and dark conditions. (-) = no response.

**Table 2:** Callus induction percentage of *P. bullata* from leaf and midrib explants cultured on woody plant medium (WPM) containing different types and concentrations of auxins under light and dark conditions.

**Light Condition**									
**Plant Growth Regulators (PGRs)**	**Leaf Explants**						**Midrib Explants**		
	**Adaxial**			**Abaxial**					
	**Callus Induction Percentage**	**Induction Time (Days)**	**Callus Morphology**	**Callus Induction Percentage**	**Induction Time (Days)**	**Callus Morphology**	**Callus Induction Percentage**	**Induction Time (Days)**	**Callus Morphology**
0	-	-	-	-	-	-	-	-	-
9.05 µM 2,4-D	-	-	-	-	-	-	-	-	-
18.10 µM 2,4-D	2.22 **^d^**	101	Compact	-	-	-	-	-	-
27.14 µM 2,4-D	-	-	-	-	-	-	2.22 **^d^**	111	Compact
36.19 µM 2,4-D	-	-	-	-	-	-	-	-	-
45.24 µM 2,4-D	-	-	-	-	-	-	-	-	-
8.28 µM picloram	-	-	-	-	-	-	11.11 **^cd^**	82	Friable
16.56 µM picloram	2.22 **^d^**	90	Friable	-	-	-	-	-	-
24.85 µM picloram	-	-	-	4.34 **^d^**	80	Friable	-	-	-
33.13 µM picloram	2.22 **^d^**	78	Friable	4.34 **^d^**	76	Friable	2.22 **^d^**	67	Friable
41.41 µM picloram	-	-	-	-	-	-	-	-	-
10.74 µM NAA	-	-	-	-	-	-	-	-	-
21.48 µM NAA	-	-	-	-	-	-	-	-	-
32.22 µM NAA	-	-	-	-	-	-	-	-	-
42.96 µM NAA	-	-	-	-	-	-	-	-	-
53.70 µM NAA	-	-	-	-	-	-	2.22 **^d^**	90	Friable
9.05 µM dicamba	-	-	-	-	-	-	-	-	-
18.10 µM dicamba	-	-	-	-	-	-	-	-	-
27.14 µM dicamba	-	-	-	-	-	-	-	-	-
36.19 µM dicamba	-	-	-	-	-	-	-	-	-
45.24 µM dicamba	-	-	-	-	-	-	-	-	-
**Dark Condition**									
**Plant Growth Regulators (PGRs)**	**Leaf Explants**						**Midrib Explants**		
	**Adaxial**			**Abaxial**					
	**Callus Induction Percentage**	**Induction Time (Days)**	**Callus Morphology**	**Callus Induction Percentage**	**Induction Time (Days)**	**Callus Morphology**	**Callus Induction Percentage**	**Induction Time (Days)**	**Callus Morphology**
0	-	-	-	-	-	-	-	-	-
9.05 µM 2,4-D	2.22 **^d^**	117	Compact	13.33 **^cd^**	117	Compact	15.55 **^bcd^**	117	Friable
18.10 µM 2,4-D	-	-	-	4.34 **^d^**	102	Compact	4.34**^d^**	102	Friable
27.14 µM 2,4-D	-	-	-	-	-	-	-	-	-
36.19 µM 2,4-D	-	-	-	-	-	-	-	-	-
45.24 µM 2,4-D	-	-	-	-	-	-	-	-	-
8.28 µM picloram	2.22 **^d^**	100	Friable	-	-	-	11.11 **^cd^**	100	Friable
16.56 µM picloram	-	-	-	-	-	-	53.33 **^a^**	78	Friable
24.85 µM picloram	-	-	-	-	-	-	13.33 **^cd^**	80	Friable
33.13 µM picloram	-	-	-	-	-	-	-	-	-
41.41 µM picloram	-	-	-	-	-	-	-	-	-
10.74 µM NAA	-	-	-	-	-	-	4.34 **^d^**	103	Friable
21.48 µM NAA	-	-	-	-	-	-	-	-	-
32.22 µM NAA	-	-	-	-	-	-	-	-	-
42.96 µM NAA	-	-	-	-	-	-	-	-	-
53.70 µM NAA	-	-	-	-	-	-	-	-	-
9.05 µM dicamba	-	-	-	-	-	-	-	-	-
18.10 µM dicamba	-	-	-	-	-	-	-	-	-
27.14 µM dicamba	-	-	-	-	-	-	-	-	-
36.19 µM dicamba	-	-	-	-	-	-	-	-	-
45.24 µM dicamba	-	-	-	-	-	-	-	-	-

Different letters indicate the values of significant difference at *p* ≤ 0.05 Tukey’s range test for light and dark conditions. (-) = no response.

**Table 3:** Total phenolic (TPC) and total flavonoid (TFC) contents of *P. bullata* callus grown on MS + 30 µM dicamba. Values are expressed as means ± SE (n = 3).

TPC (mg GAE/g FW)	TFC (mg QE/g FW)
0.657 ± 0.07	0.491 ± 0.03

**Table 4:** Pearson’s correlation coefficients between TPC and TFC with DPPH scavenging activity.

	TPC (mg GAE/g FW)	TFC (mg QE/g FW)
DPPH (%)	0.869 *	0.904 *

* Significantly different at *p* ≤ 0.05.
